# The role of CDK in the initiation step of DNA replication in eukaryotes

**DOI:** 10.1186/1747-1028-2-16

**Published:** 2007-06-05

**Authors:** Seiji Tanaka, Yon-Soo Tak, Hiroyuki Araki

**Affiliations:** 1 Division of Microbial Genetics, National Institute of Genetics, Research Organization of Information and Systems, Mishima, Shizuoka, Japan; 2 Department of Genetics, SOKENDAI, Mishima, Shizuoka, Japan; 3 Department of Biological Sciences, KAIST, Daejeon, Korea; 4 CREST, Kawaguchi, Saitama, Japan

## Abstract

Cyclin-dependent kinases (CDKs) regulate the progression of the cell cycle in eukaryotes. One of the major roles of CDK is to promote chromosomal DNA replication. However, how CDKs promote DNA replication has been a long-standing question, because all the essential CDK substrates in DNA replication have not been identified yet. Recently Sld2 and Sld3 were identified as essential substrates of CDKs in the initiation step of DNA replication in budding yeast. Moreover, bypass of their phosphorylations is sufficient to promote DNA replication. Phosphorylation of Sld2 and Sld3 by CDKs enhances the formation of complex(es) with a BRCT (BRCA1 C-Terminal)-containing replication protein, Dpb11. We further propose that multiple phosphorylation by CDKs controls this process in budding yeast. Even though Sld3 orthologues in multicellular eukaryotes have not been identified, similar complex formation and, therefore, a similar mechanism of initiation control might be employed in eukaryotes.

## Background

**Figure 1:**
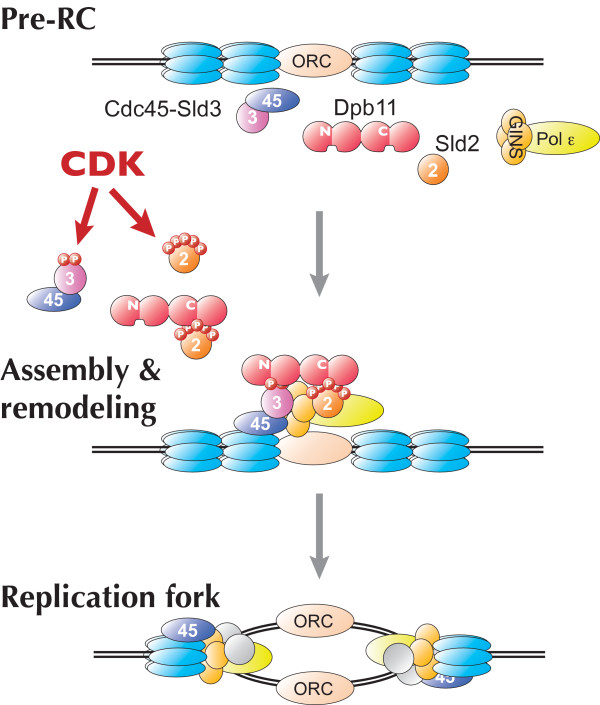
A model for CDK-regulated initiation of chromosome DNA replication. Pre-replicative complexes are formed at origins of DNA replication in G1 (top). When S-CDK is activated, it phosphorylates Sld2 and Sld3. These phosphorylations promote complex formation between Sld2 and Sld3 and Dpb11. This reaction triggers the initiation of DNA replication.

**Figure 2:**
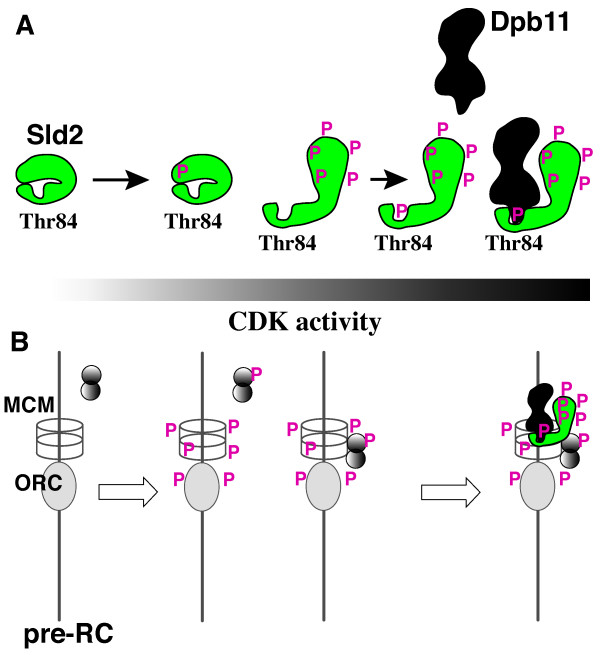
Regulatory model of the interaction between Dpb11 and Sld2 phosphorylated by CDK. (**A**) The phosphorylation level of Sld2 is proportional to the level of CDK activity. However, phosphorylation of Thr84 in Sld2 requires prior phosphorylation of other CDK phosphorylation motifs. When CDK activity increases beyond threshold, Sld2 may change its conformation by multiple phosphorylations and then CDK phosphorylates Thr84. When Thr84 is phosphorylated, Sld2 forms a complex with Dpb11 to initiate DNA replication. (**B**) The pre-replicative complex (pre-RC) essential for the initiation of chromosomal DNA replication is formed at replication origins from late M phase to G1 phase when CDK activity is low. When CDK activity increases at G1/S phase boundary, the pre-RC components are phosphorylated and inactivated for further formation of the pre-RC before Thr84 of Sld2 is phosphorylated. Some other proteins are also phosphorylated and may bind to origins before Thr84-phosphorylation. Thus, inactivation of the pre-RC formation and preceding origin association of some replication proteins are ensured by this mechanism.

It has been known for a long time that CDK activity is required for DNA replication in eukaryotes. This concept was established by the early 1990s. For example, CDK-containing fractions activated the replication of SV40 virus in cellular extracts [1] ; Immunodepletion of CDKs prevented the initiation of DNA replication in vitro in Xenopus egg extracts [2]; and finally, CDK or cyclin mutants cannot promote DNA replication in yeasts [3,4]. From that time the hunt for CDK targets became to a major issue in the research of DNA replication. We and others recently revealed that phosphorylation of two replication proteins, Sld2 and Sld3, is essential and minimal requirement for CDK-dependent activation of the initiation of DNA replication in budding yeast (Fig. 1) [5,6]. We also proposed that highly regulated phosphorylation of Sld2 helps fine-tuning of the initiation (Fig. 2) [7]. In this review, we will summarize recent findings and envision future direction of these studies.

Chromosomal DNA replication in eukaryotes initiates from multiple replication origins. Activities of individual origins are regulated as follows: The six-subunit origin recognition complex (Orc) binds to replication origins throughout the cell cycle in yeast cells, and the putative replicative helicase Mcm (Mcm2-7 complex) is recruited onto Orc-bound origins with the aid of Cdc6 and Cdt1 to form the pre-replicative complex (pre-RC), from late M to G1 phase when CDK activity is low. Once both CDK and Dbf4-dependent protein kinase (DDK/Cdc7), another protein kinase essential for DNA replication, are activated, many replication proteins including replicative DNA polymerases, α, δ and ε are loaded onto the pre-RC formed origins. Then origin DNA is unwound and replication forks are formed to synthesize DNA (see reviews; [8,9]). For DNA replication after the initiation, both CDK and DDK are dispensable [2,10,11], our unpublished results].

## CDK-targets at the initiation step of DNA replication

We previously showed that budding-yeast Sld2 is an essential CDK target for DNA replication [12]. The *sld2 *mutation was originally isolated as one of *sld *(synthetically lethal with *dpb11-1*) [13]. The Sld2 protein has a cluster of 11 CDK phosphorylation motifs (Ser/Thr-Pro). At one of these motifs, phosphorylation of Thr84 promotes an essential complex formation between Sld2 and another replication protein, Dpb11, while Thr84-phosphorylation requires other phosphorylations in Sld2 (see below) [7]. Dpb11 has two pairs of tandem BRCT (BRCA1 C-Terminal) domains, which is known as a phosphopeptide-binding domain [14,15]. C-terminal pair of BRCT domains in Dpb11 binds to phosphoThr84-Sld2 [7]. Dpb11 and Sld2 associate with replication origins when Cdk1 (Cdc28 in budding yeast) is activated [16], our unpublished results]. However, phosphorylation of Sld2 alone does not promote initiation of DNA replication. A phosphomimetic form of Sld2 constructed by substituting aspartic acid for serine or threonine residue in CDK phosphorylation motif could support cell growth but Cdk1 was still required for initiation [6]. This strongly suggests the existence of other targets of S-phase CDK at the initiation step of DNA replication.

Actually, we and others found another essential CDK target, Sld3 [5,6]. The *sld3 *mutation was also isolated by the '*sld*' screening [13]. Of 12 CDK phosphorylation motifs in Sld3, phosphorylations at Thr600 and Ser622 are crucial for binding Sld3 to N-terminal pair of tandem BRCT domains in Dpb11. Simultaneous alanine substitutions (Sld3-2A) and any combination of aspartic acid and glutamic acid substitutions for these residues confer cell lethality and defects of DNA replication, suggesting that phosphorylations of Thr600 and Ser622 are essential for cell growth and DNA replication and aspartic acid and glutamic acid residues do not mimic a phosphorylated residue at these sites. However, there are three ways to restore growth of *sld3-2A *mutant cells. First, multicopy *DPB11 *suppresses lethality caused by *sld3-2A *[6]. Overexpression of binding partners often suppresses a mutation occurring in the other subunit. This is the case for Dpb11 and Sld3. Second, the *JET1-1 *mutation (Jumping Essentiality of CDK with sld2(Two)-11D to initiate DNA replication) restores the growth defect [6]. *JET1-1 *was isolated as a mutation, which induces untimely DNA replication when the phosphomimetic Sld2 (Sld2-11D) was expressed. The *JET1-1 *mutation occurred in the *CDC45 *gene, whose product binds to Sld3. However, Cdc45 itself is not a target of Cdk1 in the initiation, because disruption of all the CDK phosphorylation motifs in Cdc45 does not show any defect in DNA replication nor cell growth. Jet1-1 seems to enhance the interaction between Sld3 and Dpb11 through the interaction between Cdc45 and Sld3 [6]. Third, the fusion protein between Sld3-2A and C-terminal half of Dpb11 (SD fusion) can replace Sld3 and Dpb11 simultaneously because N-terminal pair of Dpb11 functions as the binding site to Sld3 and SD fusion functions as Sld3-Dpb11 complex[5]. These results imply that phosphorylation-dependent interaction between Dpb11 and Sld3 is CDK-regulated essential step for the initiation of DNA replication.

## Phosphorylation of Sld2 and Sld3 is sufficient for the initiation of DNA replication

We combined *JET1-1*, multicopy *DPB11 *and the *sld2-D *mutation while Zegerman and Diffley combined the SD fusion and the *sld2-D *mutation [5,6]. In any case, the combinatory strains initiated DNA synthesis even when lacking S-phase CDK activities. DNA synthesis observed in the absence of CDK activity reflects DNA replication by several criteria. In normal DNA replication, two protein kinases, CDK and DDK, activate DNA replication. Both combinatory strains require DDK activity to initiate DNA synthesis, indicating that the cells only bypass CDK requirement in the initiation. Furthermore, semi-conservative DNA synthesis initiates from known replication origins in a pre-RC-dependent manner with proper replication fork complex, CMG (Cdc45-Mcm-GINS) in *JET1-1 sld2-D *mutant cells lacking CDK activity (DNA synthesis in SD fusion strain has not been characterized in detail). In any combinatory strains, DNA replication induced in G1 cells is not efficient as that in CDK-driven DNA replication. Nonetheless, these data strongly suggest that phosphorylation of Sld2 and Sld3 by Cdk1 are the minimal requirement for the initiation of DNA replication although we cannot rule out the formal possibility that the SD fusion and *JET1 *bypass not only Sld3 phosphorylation but also other phosphorylations.

## CDK-regulated DNA replication

While phosphorylation-dependent interactions between Sld2, Sld3 and Dpb11 appear to be essential to initiate DNA replication, how these interactions facilitate initiation is still obscure. A novel complex, pre-Loading Complex (pre-LC) containing Polε, GINS, Dpb11 and Sld2 was detected in the cells treated with a cross-linking reagent and this complex formation depends on CDK but not DDK nor pre-RC (our unpublished results). On the other hand, Sld3, together with Cdc45, associates with pre-RC-formed early-firing origins even in G1 phase [17]. Thus, when Cdk1 is activated, Sld3 on origins is phosphorylated and binds to Dpb11, which might be a component of the pre-LC (Fig. 1). While we do not know whether Dpb11 binds to Sld2 and Sld3 in a sequential or random manner, and whether a single Dpb11 molecule binds to Sld3 and Sld2 simultaneously, these interaction may contribute to association between proteins bound to Sld2, Sld3 and Dpb11, such as GINS, Cdc45 and Mcm. Recent studies have identified complexes containing Cdc45, Mcm and GINS in Xenopus, budding yeast, and Drosophila, which are suggested to work at replication forks [18-20]. Moreover, the CMG complex composed of these three factors from Drosophila shows helicase activity, suggesting that it works as a replicative helicase [19]. Thus, we suggest CDK-phosphorylation dependent interactions between Dpb11, Sld2 and Sld3 may play a role in the formation of CMG complex.

## Fine-tuning by multisite phosphorylation

The phosphorylation-dependent interaction between Dpb11, Sld2 and Sld3 may be an essential mechanistic requirement to promote the initiation of DNA replication. It is also conceivable that these interactions monitor cellular CDK activity to couple DNA replication with other cell cycle events. We will discuss the interaction between Dpb11 and Sld2 from our viewpoint.

The peptide corresponding to residues 79–107 of Sld2 binds to the C-terminal pair of tandem BRCT domains in a Thr84-phosphorylation dependent manner. However, disruption of other CDK phosphorylation sites of Sld2 abolishes the interaction between Dpb11 and Sld2 [7]. This is because phosphorylation at Thr84 requires prior phosphorylation of other sites by Cdk1. Thus, phosphorylation of Thr84 occurs later than other phosphorylations sites and consequently multisite phosphorylation set a high threshold for CDK activity to form complex between Dpb11 and Sld2. Conversion of the CDK phosphorylation motif, Thr^84^-Pro, to PIKK (PI3 kinase related kinase) phosphorylation motif, Thr^84^-Gln, results in phosphorylation of Thr84 by DNA-dependent protein kinase, one of PIKK, and this phosphorylation depends on pre-phosphorylation by CDK. We thus argue that phosphorylation renders Thr84 accessible to DNA-dependent kinase in the Thr-Gln construct and to Cdk1 in the original construct, probably by phosphorylation-dependent conformational change (Fig. 2).

The high threshold of CDK activity may prevent premature replication. When Cdk1 is activated and DNA replication initiates, the pre-RC is converted to post-replicative complex (post-RC) composed of Orc at the replication origins. Active Cdk1 phosphorylates the pre-RC components and inhibits reassembly of pre-RC to prevent reinitiation (reviewed by [21]). The high threshold of CDK activity for Thr84 phosphorylation ensures complete phosphorylation of the pre-RC components before DNA replication initiates.

The Sld3 protein has 12 CDK phosphorylation motifs. Two of them, Thr600 and Ser622, are responsible for CDK phosphorylation-dependent interaction between Dpb11 and Sld3 [5,6]. Simultaneous alanine substitution at all the CDK phosphorylation motifs except Thr600 and Ser622 does not affect cell growth, suggesting that phosphorylation of Sld3 does not affect phosphorylation of Thr600 and Ser622, unlike in Sld2. On the other hand, efficient binding of Sld3 to N-terminal pair of BRCT domains in Dpb11 requires simultaneous phosphorylations of Thr600 and Ser622, which may require high activity of Cdk1. Further investigation will reveal the precise regulation of phosphorylation-dependent interaction between Sld3 and Dpb11 and how Sld2 and Sld3 bind to Dpb11, for example sequential or random.

## Regulation of initiation in other organisms

Because regulation of DNA replication as well as the cell cycle seems to be well conserved throughout eukaryotes, the molecular mechanisms of the initiation of DNA replication regulated by CDK might be conserved. Orthologues of Sld2, Sld3 and Dpb11 are found in yeast and fungi while in other organisms they seem to differ significantly (Tables 1, 2 and 3). In animal cells, TopBP1/Cut5/Mus101 (Table 1) is thought as a counterpart of Dpb11 because they have seven or eight BRCT domains in animals and is required for DNA replication (reviewed in [22]). Moreover, TopBP1/Cut5/Mus101 binds to Xenopus RecQL4 (Table 2), which has a limited homology to Sld2 and is required for DNA replication, while phosphorylation-dependent interaction has not been observed [23]. Sld3 seems to be most diverse, because we cannot find a protein similar to Sld3 except in yeast and fungi (Table 3) [24]. We believe that functional homologue of Sld3 exists and functions in DNA replication. Plant cells have small number of multiple BRCT-containing proteins and we cannot identify Dpb11, Sld2 and Sld3 homologues although they have a conserved replication machinery, such as pre-RC, Cdc45, GINS and DNA polymerases, and CDKs. This evidence suggests that plant cells employ phosphorylation-dependent interaction different from BRCT phosphopeptide domains.

**Table 1:** Dpb11 orthologues

Species	Gene product	Features
*S. cerevisiae*	Dpb11	4x BRCT. N-terminal and C-terminal pair binds phosphorylated Sld3 and phosphorylated Sld2, respectively. [6, 12, 14]
*S. pombe*	Cut5/Rad4	4x BRCT. N-terminal pair is shown to bind Drc1. [25, 26]
*C. elegans*	Mus101	6x BRCT. Required for DNA replication. MMS sensitibity by RNAi feeding. SUMO modification? [27]
*D. melanogaster*	Mus101	7x BRCT. Involvement in DNA replication is suggested. [28, 29]
*X. laevis*	Cut5/Mus101	8x BRCT. Functions in DNA replication and DNA replication or damage checkpoints. [30–32]
*H. sapiens*	TopBP1	8x BRCT. Originally isolated as topoisomerase-binding protein. Functions in DNA replication and DNA replication or damage checkpoints. [33–35]

**Table 2:** Sld2 orthologues

Species	Gene product	Features
*S. cerevisiae*	Sld2/Drc1	Phosphorylation at T84 by CDK is essential for initiation. Phosphorylated Sld2 binds Dpb11 C-terminus. Alanine-substitution mutant is lethal. [7, 12]
*S. pombe*	Drc1	Phosphorylation by CDK is essential for initiation. Alanine-substitution mutant is lethal. [26]
*X. laevis*	RTS/RecQ4	N-terminal portion show similarity to Sld2. C-terminal portion has RecQ helicase motif. It binds Cut5 and is required for DNA replication. [36]
*H. sapiens M.musculus*	RTS/RecQL4	N-terminal portion show similarity to Sld2. C-terminal portion has RecQ helicase motif. It is essential for cell proliferation. Rothmund-Thomson syndrome and Rapadilino syndrome cells have mutaions in this gene. [36]

**Table 3:** Sld3 orthologues

Species	Gene product	Features
*S. cerevisiae*	Sld3	Binds to Cdc45, GINS and Dpb11. Phosphorylation at T600 and S622 by CDK is essential for initiation. Phosphorylated Sld3 binds Dpb11 N-terminus. Alanine-substitution mutant is lethal. [5, 6]
*S. pombe*	Sld3	Required for initiation (Cdc45 loading). Chromatin loading of Sld3 depends on DDK but not CDK. [24]

## Conclusion

In budding yeast Cdk1 promotes chromosomal DNA replication by phosphorylating two replication proteins, Sld2 and Sld3. Phosphorylated form of Sld2 and Sld3 bind to BRCT-containing protein, Dpb11 and these interactions are essential for initiating DNA replication. Bypassing these phosphorylation-dependent interactions by various ways promotes DNA replication in the absence of CDK activity. Thus, we conclude that CDK-dependent phosphorylations of Sld2 and Sld3 and consequently their interactions with Dpb11 are minimal requirements for CDK-dependent activation of the initiation of DNA replication. Furthermore, multiple phosphorylation of Sld2, at least, contributes to fine-tuning of this step. Although Dpb11, Sld2 and Sld3 are not well conserved in multi-cellular organisms, CDK may regulate the initiation step of DNA replication in a similar manner, that is, phosphorylation-dependent interactions of replication proteins.

## Competing interests

The author(s) declare that they have no competing interests.

## Authors' contributions

All the authors wrote the manuscript and H.A. organized it.
